# Self-reported body function and daily life activities 18 months after Covid-19: A nationwide cohort study

**DOI:** 10.1177/14034948241272949

**Published:** 2024-09-18

**Authors:** Johanna Seljelid, Annie Palstam, Katharina S. Sunnerhagen, Hanna C. Persson

**Affiliations:** 1 Department of Clinical Neuroscience, Institute of Neuroscience and Physiology, Sahlgrenska Academy, University of Gothenburg, Sweden; 2 Department of Rehabilitation medicine, Sahlgrenska University Hospital, Gothenburg, Sweden; 3 School of Health and Welfare, Dalarna University, Sweden; 4 Department of Occupational Therapy and Physical Therapy, Sahlgrenska University Hospital, Sweden

**Keywords:** Covid-19, body function, daily life activities, long term, sick leave, surveys and questionnaires, International Classification of Functioning, Disability and Health, sex differences

## Abstract

**Aims:**

This study aimed to investigate body function and daily life activities 18 months after Covid-19 infection, depending on the initial severity of disease and according to sex.

**Methods:**

All 11,955 individuals on sick leave due to Covid-19 during the first wave of the pandemic in Sweden were invited to answer a questionnaire regarding experiencing negative changes in body function and daily life activities approximately 18 months after the start of sick leave. The analysis of data included descriptive statistics, group comparisons and multivariable binary logistic regressions (two groups).

**Results:**

Of 5464 responders (45.7%), 4676 (85.6%) reported experiencing at least one problem with body function, and the reported prevalence of problems with daily life activities was 46%. The most frequently reported problems were fatigue (66.3%), cognition, sleep and movement. In general, women and those initially hospitalised reported more problems. In the regression analyses, problems with body function could partly explain whether individuals experienced problems with daily life activities. However, only fatigue and movement significantly contributed throughout all groups (*p*<0.001). Furthermore, the odds ratios for fatigue were larger in regressions for women than for men.

**Conclusions:**

**In this nationwide study, more than 8 out of 10 individuals experienced problems with body function 18 months after being on sick leave due to Covid-19, with women and those initially hospitalised reporting more problems. Problems with body function, such as fatigue, could partly explain problems with daily life activities. However, the mechanisms behind the consequences are not yet clear and need to be further investigated.**

## Introduction

Since the start of the pandemic in 2019, more than 775 million cases of Covid-19 infection have been reported globally, including more than 2.7 million in Sweden [1]. The pandemic has affected all aspects of public health, including the determinants of health [2], due to both the infection itself and the restrictions and recommendations applied to minimise the spread of infection. The long-term consequences of Covid-19 are not yet fully understood. However, they risk affecting not only the individual but also health care and other social institutions, including social insurance.

During the first wave of the pandemic in Sweden (1 March to 31 August 2020), new cases of sick leave doubled compared to the same period the previous year, with women accounting for 60% of these cases [3]. Nearly 12,000 people in Sweden were on sick leave due to Covid-19 during the first wave, as previously investigated by our research group [4,5], and they constitute the cohort for the current study. Approximately 13% of the cohort was reported to be on sick leave for at least 12 weeks, and the strongest predictor of longer sick leave was hospitalisation due to Covid-19, followed by older age, sick leave the year before Covid-19 and male sex [4]. Recurrent sick leave was more common among women, older people and those who had been on sick leave the year before Covid-19 [5].

A wide range of symptoms have previously been reported as long-term consequences of Covid-19 [6–8], and among the most commonly reported symptoms in the first year are problems with fatigue, pain, sleep, breathing and daily life activities [9]. However, as the symptoms are diverse [10], follow-up studies of Covid-19 could benefit from a framework such as the International Classification of Functioning, Disability, and Health (ICF) to measure outcomes more coherently [11]. Symptoms related to the long-term consequences of Covid-19 are also relatively prevalent in the general population [12]. Several studies have, however, adjusted for this and reported a higher prevalence of symptoms in individuals who have had Covid-19 [12–14], suggesting causality.

The prevalence of long-term consequences varies among studies [12–16], and comparisons are complicated by the heterogeneity of studies regarding study population, follow-up time and the symptoms assessed [10]. Factors reported to be associated with a higher risk of long-term consequences are older age, female sex and more severe disease [12]. Vaccination has been reported as a protective factor [17].

For those who experience long-term consequences of Covid-19, studies report protracted recoveries [12,18]. Together with the large numbers affected by Covid-19 and the relatively high prevalence of long-term consequences, this implies a great challenge for individuals and society as a whole. Larger populations and longer follow-ups are needed to fully understand the scope and better help the people affected.

## Aims

The aim of this study was to investigate health status in regard to body function and daily life activities 18 months after Covid-19 infection, depending on the initial severity of the disease and patient sex using self-reported data from a Swedish national cohort of individuals who were on sick leave due to Covid-19 during the first wave of the pandemic.

## Methods

### Study design and population

This is a national cohort survey of self-reported health 18 months after Covid-19 infection in Sweden. The study comprises a register-based population with data from the Swedish Social Insurance Agency (SSIA), the National Board of Health and Welfare, and Statistics Sweden. In Sweden, sick leave is managed by the SSIA, with registries including employees absent from work for 15 days or more and self-employed or unemployed staying home for one day or more based on regulation of sickness benefits. All individuals in Sweden registered as being on sick leave due to Covid-19 at the SSIA, defined by International Statistical Classification of Diseases (ICD) code U07 (U07.1: Covid-19, virus identified; U07.2: Covid-19, virus not identified) as the main or secondary diagnosis, between 1 March and 31 August 2020 (first wave) were included in the cohort. The study combines long-term follow-up survey data and data from national registers.

### Data collection procedures

Approximately 18 months after the start of sick leave, a questionnaire was sent out to everyone included in the cohort. The questionnaire enquired about Covid-19 in the acute phase and current health status and was constructed based on the knowledge of long-term consequences of Covid-19 at the time, including the literature, a follow-up survey used after hospital care available at the Swedish Healthcare Guide online (1177.se) and guidelines for assessment during the follow-up phase, as produced by a national working group consisting of medical experts, the National Board of Health and Welfare, and a patient organisation [19]. Questions were categorised according to the ICF.

The survey was administered by Indicator (Institutet för kvalitetsindikatorer). Dispatches were sent out on 25 February and 16 March 2022, with the invitation, study description, conditions of participation and the questionnaire in paper format. Informed consent was given at the time that the survey was answered. Participants could answer online or by mail, and reminders were sent out by text message on 8 March and 30 March 2022. The study was approved by the Swedish Ethical Review Authority on 24 June 2020 (2020-03046), with an amendment approved on 1 September 2021 (2021-03556).

### Variables

Data on sex, age, education level and country of birth were collected from the National Board of Health and Welfare and Statistics Sweden. Education level was presented as primary school (⩽9 years), secondary school (10–12 years), short university education (13–14 years) and long university education (⩾15 years). Country of birth was categorised into Sweden, European countries except for Sweden and countries outside of Europe. Data on the length of sick leave and sick leave prior to Covid-19 were collected from the SSIA. Length of sick leave was dichotomised, with the cut-off set to three months (0–90 days/>90 days), in line with the World Health Organization definition of post-Covid. Sick leave prior to Covid-19 was defined as having been on sick leave for 28 days or more or six times or more the year prior to Covid-19. Covid-19 severity was categorised as hospitalised (more severe) or non-hospitalised (less severe), referring to whether individuals were hospitalised during their Covid-19 infection, with data from the National Board of Social Affairs and Health.

Variables defined as body function were movement, pain and touch perception, symptoms of anxiety or depression, cognition, anosmia or ageusia, swallowing, sleep, fatigue, voice, breathing, cough, heart rhythm, skin and temperature alterations. Problems with body function and daily life activities (i.e. showering, shopping, social activities, etc.) were defined as answering ‘yes’ (yes/no) on and/or grading (small, some or big problems) questions enquiring about perceived negative changes since Covid-19 in regard to the specified body function and daily life activities.

### Statistical analysis

Statistical analyses were performed using IBM SPSS Statistics for Windows v29.0 (IBM Corp., Armonk, NY) and R v4.3.2 (The R Foundation for Statistical Computing, Vienna, Austria). A *p*-value of <0.05 was considered significant unless stated otherwise. A non-response analysis was performed using the Mann–Whitney *U*-test for age, educational level and length of sick leave; the chi-square test for sex, country of birth, Covid-19 severity, and sick leave of more than three months; and the independent *t*-test for days on sick leave. The prevalence of problems with body function and daily life activities (yes/no) was analysed using descriptive statistics, presented in absolute numbers and percentages, and compared depending on sex (male/female) and Covid-19 severity using the chi-square test. The grading of problems with body function was presented in 100% stacked bar charts as ordinal variables (no, small, some or big problems).

Explanatory factors for problems with daily life activities were assessed with multivariable binary logistic regression. The dependent variable was problems with daily life activities (yes or no) and, if yes, thereafter rated on a scale which was dichotomised into two categories ‘no-small problems’ and ‘some-big problems’. If there were missing values in the grading, but with a positive response in the question regarding problems with daily life activities, this was categorised as problem present, category ‘some-big’. Previous research on the same population have pointed out differences in length of sick leave, depending on Covid-19 severity [4,5] and sex [4]. Therefore, we chose to report the results according to the findings, stratified by hospitalisation (as a proxy for Covid-19 severity) and sex, respectively. The logistic regression model was fitted separately for men and women. It included hospitalisation (yes/no) as both a main effect and in the form of all pairwise interactions of this variable with all other explanatory variables. Explanatory variables were chosen based on clinical relevance and included the following: age, education level, sick leave prior to Covid-19, sick-leave length and grading of acute symptoms, movement, cognition, fatigue and breathing. Explanatory variables were controlled for multicollinearity using the chi-square test and Spearman’s rank correlation, with significance set to *p*<0.05 and Spearman’s rank–order correlation (RHO) >0.7, respectively. In the case of multicollinearity, the least clinically relevant variable was excluded from further analysis. To ensure sufficient quantity in each category (five or more) of explanatory variables, they were controlled against the dependent variable using cross tabs; if there were fewer than five, the categories were collapsed. In the regression, body functions were analysed as ordinal variables (no, small, some or big problems). The results are presented in forest plots, with odds ratios (ORs), 95% confidence interval (CIs) and *p*-values for each regression, including *p*-values with Bonferroni correction. The R package rms v6.7-1 (https://CRAN.R-project.org/package=rms) was used for the regression analysis [20]. Results of the regression analysis with no interaction are available in the supplemental material.

## Results

Of 11,955 individuals included in the cohort, 5464 (45.7%) responded to the questionnaire approximately 18 months after the start of sick leave. There was a significant difference between responders and non-responders regarding all characteristics evaluated (Table I). Responders included a larger proportion of women (65.9%), individuals born in Sweden (76.6%), individuals with long and short university education, and individuals who were initially hospitalised due to Covid-19 (27.5%) than non-responders. Responders were also older and had longer sick leave (median 36 days). Within responders, men comprised 59% of hospitalised individuals, and women comprised 75.3% of non-hospitalised individuals.

**Table I.** Characteristics of the cohort.

	Total	Responders	Non-responders	*p*-Value
Participants, *n* (%)	11,955 (100)	5464 (45.7)	6491 (54.3)	
Sex, *n* (%)				<0.001*
Male	4828 (40.4)	1865 (34.1)	2961 (45.6)	
Female	7129 (59.6)	3599 (65.9)	3530 (54.4)	
Age, years, *M* (*SD*)	48.0 (11.3)	50.9 (10.2)	45.6 (11.6)	<0.001**
Country of birth, *n* (%)^a^				<0.001*
Sweden	7545 (63.2)	4182 (76.6)	3362 (51.9)	
European countries except for Sweden	1481 (12.4)	509 (9.3)	972 (15.0)	
Countries outside of Europe	2920 (24.4)	772 (14.1)	2148 (33.1)	
Education level, *n* (%)^b^				<0.001**
Primary school (⩽9 years)	1237 (10.4)	347 (6.4)	890 (13.9)	
Secondary school (10–12 years)	5889 (49.6)	2598 (47.6)	3291 (51.3)	
Short university education (13–14 years)	1743 (14.7)	841 (15.4)	902 (14.1)	
Long university education (⩾15 years)	2995 (25.2)	1667 (30.6)	1328 (20.7)	
Covid-19 severity, *n* (%)				<0.001*
Non-hospitalised	8995 (75.2)	3961 (72.5)	5034 (77.6)	
Hospitalised	2960 (24.8)	1503 (27.5)	1457 (22.4)	
Sick-leave length, days				
*M* (*SD*)	62.1 (83.0)	67.3 (91.8)	57.8 (74.5)	<0.001***
Median (IQR)	35 (29)	36 (28)	35 (24)	0.003**
Sick-leave length >3 months, *n* (%)	1451 (12.1)	762 (13.9)	689 (10.6)	<0.001*

The *p*-value refers to the difference between responders and non-responders.

a 9 missing; ^b^91 missing.

* Chi-square test; **Mann–Whitney *U*-test; ***independent *t*-test.

*SD*: standard deviation; IQR: interquartile range.

Of 5464 responders, 4676 (85.6%) reported experiencing at least one problem with body function (Table II). The mean number of reported problems with body function was five (*M*=5.2, *SD*=3.9; median 5, interquartile range=6). Fatigue was the most frequently reported problem (66.3%), followed by cognition (55.0%), sleep (51.6%) and movement (50.3%). The lowest prevalence reported for any problem with body function was 16.9% for swallowing.

**Table II.** Prevalence and differences in problems with body function.

Problems with:	Total, *n* (%)	Men, *n* (%)	Women, *n* (%)	*p*-Value	Non-hospitalised, *n* (%)	Hospitalised, *n* (%)	*p*-Value
⩾1 body function^a^	4676 (85.6)	1558 (83.5)	3117 (86.6)	0.002	3358 (84.8)	1317 (87.6)	0.007
Movement^b^	2655 (50.3)	932 (51.5)	1723 (49.6)	0.185	1840 (48.2)	815 (55.7)	<0.001
Pain and touch perception^c^	1957 (37.1)	621 (34.3)	1336 (38.5)	0.003	1363 (35.7)	594 (40.7)	<0.001
Bowel function^d^	1276 (24.2)	370 (20.4)	906 (26.1)	<0.001	903 (23.7)	373 (25.5)	0.162
Symptoms of anxiety or depression^e^	2312 (43.7)	755 (41.7)	1557 (44.8)	0.035	1596 (41.7)	716 (48.9)	<0.001
Cognition^f^	2899 (55.0)	906 (50.2)	1993 (57.5)	<0.001	2034 (53.4)	865 (59.2)	<0.001
Anosmia/ageusia^g^	1937 (36.6)	540 (29.8)	1397 (40.2)	<0.001	1482 (38.8)	455 (31.1)	<0.001
Swallowing^h^	894 (16.9)	1809 (17.0)	586 (16.9)	0.913	589 (15.4)	305 (20.9)	<0.001
Sleep^i^	2707 (51.6)	834 (46.4)	1873 (54.3)	<0.001	1900 (50.1)	807 (55.5)	<0.001
Fatigue^j^	3513 (66.3)	1155 (63.7)	2358 (67.6)	0.004	2505 (65.4)	1008 (68.6)	0.024
Voice^k^	1179 (22.3)	355 (19.7)	824 (23.6)	<0.001	812 (21.2)	367 (25.1)	0.002
Breathing^l^	2303 (43.5)	756 (41.8)	1547 (44.4)	0.077	1546 (40.4)	757 (51.6)	<0.001
Cough^m^	1223 (23.1)	429 (23.7)	794 (22.8)	0.468	851 (22.2)	372 (25.4)	0.015
Heart rhythm^n^	1635 (30.9)	399 (22.0)	1236 (35.5)	<0.001	1221 (31.9)	414 (28.3)	0.012
Skin^o^	1047 (19.8)	270 (14.9)	777 (22.4)	<0.001	766 (20.1)	281 (19.2)	0.46
Temperature alterations^p^	999 (18.9)	200 (11.1)	799 (23.0)	<0.001	800 (21.0)	199 (13.6)	<0.001

Prevalence analysed in 5464 responders. Differences depending on sex and Covid-19 severity were analysed using the chi-square test. The *p*-values refer to the difference in prevalence between men and women and between non-hospitalised and hospitalised responders, respectively. Footnote letters indicate missing answers for the question regarding that body function: ^a^128, ^b^184, ^c^188, ^d^187, ^e^178, ^f^195, ^g^178, ^h^189, ^i^217, ^j^162, ^k^174, ^l^168, ^m^171, ^n^175, ^o^182, ^p^187.

Differences between men and women were significant in all but four of the body functions (movement, swallowing, breathing, cough), with women reporting a higher prevalence of problems (Table II). The largest differences between women and men were seen in problems with anosmia or ageusia (40.2% vs. 29.8%, *p*<0.001), heart rhythm (35.5% vs. 22.0%, *p*<0.001) and temperature alterations (23.0% vs. 11.1%, *p*<0.001).

Differences depending on Covid-19 severity were significant in all but two body functions (bowel function, skin), with hospitalised responders generally reporting a higher prevalence of problems (Table II). However, non-hospitalised responders reported a higher prevalence of problems with anosmia or ageusia, heart rhythm and temperature alterations. The largest differences between hospitalised and non-hospitalised responders were seen in problems with movement (55.7% vs. 48.2%, *p*<0.001), anosmia or ageusia (31.1% vs. 38.8%, *p*<0.001) and breathing (51.6% vs. 40.4%, *p*<0.001).

**Figure 1.**
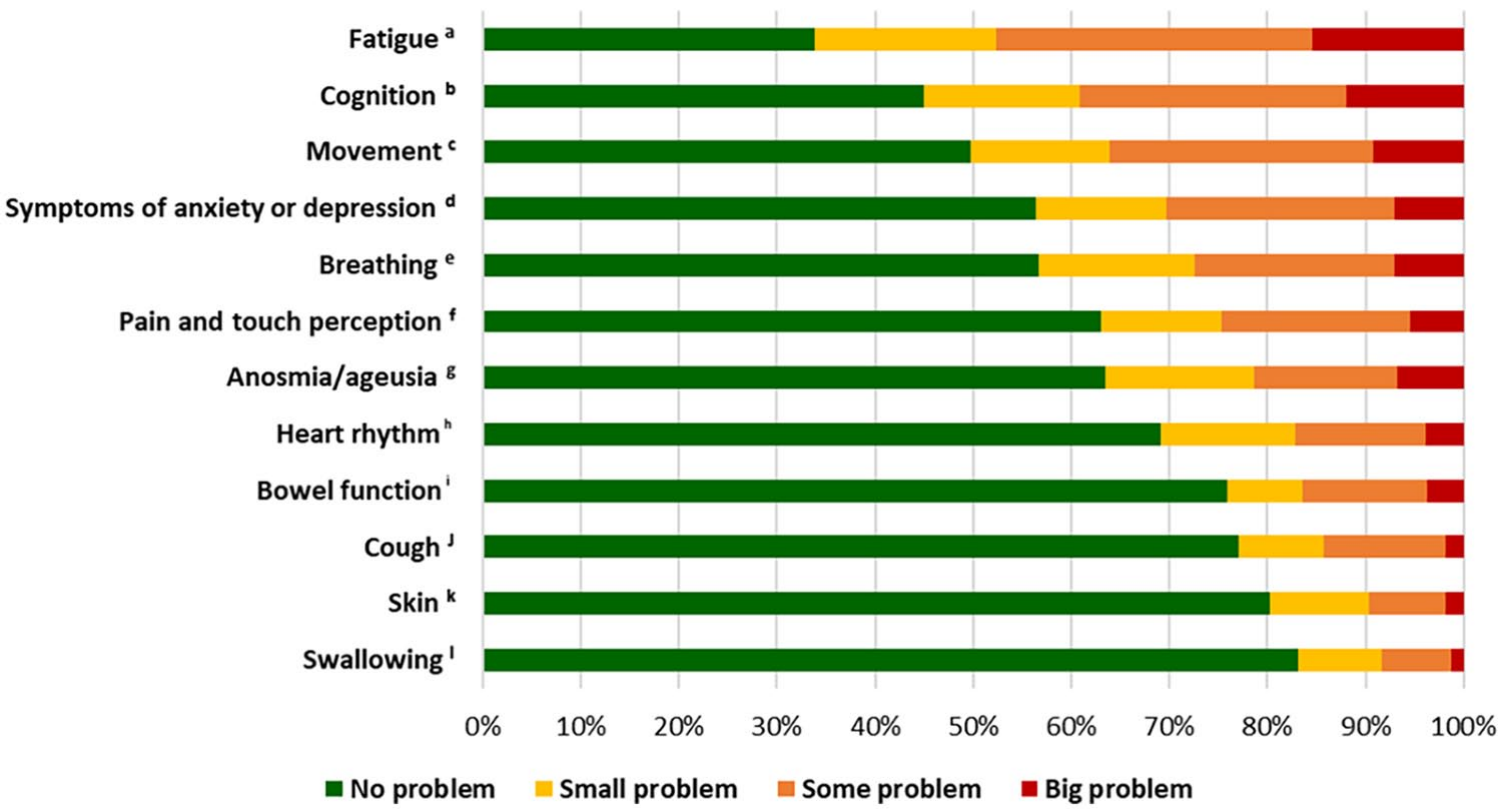
Grading of problems with body function. A four-point ordinal scale reported by 5464 people, presented in descending order with 100% stacked bars. Footnote letters indicate missing answers for the question regarding that body function: ^a^180, ^b^205, ^c^193, ^d^186, ^e^187, ^f^196, ^g^186, ^h^182, ^i^194, ^j^180, ^k^187, ^l^190.

Of the participants who reported problems with body function, most generally reported ‘some problems’ (Figure 1). However, in regard to anosmia or ageusia, swallowing, heart rhythm and skin, a larger proportion reported having a ‘small problem’.

The reported prevalence of problems with daily life activities was 46%; 9.5% reported a small problem, 26% reported some problem and 10.3% reported a big problem. A significant difference was seen depending on Covid-19 severity, with hospitalised responders reporting a higher prevalence (50.3% vs. 44.3%, *p*<0.001). In women, the reported prevalence of problems was 46.6%; in men, the reported prevalence was 44.8% (*p*=0.209).

The results of the binary logistic regression model for men are presented in Table III. Of the 10 explanatory variables, sick leave of more than three months, movement, cognition, fatigue, breathing and hospitalisation made a significant contribution to the model even after applying Bonferroni correction (10 tests, corrected significance level=0.005). Hospitalisation seems to modify how the other variables contribute to the outcome (*p*=0.0152), but this result is mainly due to the variable sick leave of more than three months (OR=9.81, 95% CI 3.82–25.2 in the non-hospitalised men and OR=1.07, 95% CI 0.64–1.79 in the hospitalised men). The effect of hospitalisation must be therefore interpreted with caution, since all the information about non-hospitalised men on sick leave for more than three months comes from only 69 individuals.

**Table III.** Explanatory factors for problems with daily life activity among men.

	Non-hospitalised men	Hospitalised men	Association of the variable as such (in at least one of the subgroups)	Difference between groups (in at least 1 OR)
Age (years)				
18–40 (ref.)			*p*=0.1558	*p*=0.2465
41–50	0.88 (0.42–1.84)	2.30 (0.78–6.77)		
51–60	1.47 (0.76–2.84)	2.82 (1.03–7.76)		
⩾60	0.77 (0.34–1.71)	2.72 (0.95–7.82)		
Education				
Primary school (ref.)			*p*=0.7159	*p*=0.8593
Secondary school	0.75 (0.31–1.78)	0.56 (0.27–1.15)		
Short university education	0.87 (0.32–2.36)	0.58 (0.25–1.34)		
Long university education	0.84 (0.33–2.14)	0.49 (0.2–1.16)		
Sick leave prior to Covid-19	0.60 (0.3–1.18)	1.60 (0.72–3.57)	*p*=0.1685	*p*=0.0644
Sick leave length >3 months	9.81 (3.82–25.2)	1.07 (0.64–1.79)	*p*<0.0001	*p*<0.0001
Acute symptoms				
No moderate problems (ref.)			*p*=0.6898	*p*=0.9723
Quite severe problems	1.10 (0.64–1.86)	1.13 (0.4–3.23)		
Very severe problems	1.51 (0.74–3.08)	1.41 (0.51–3.88)		
Movement				
No problem (ref.)			*p*<0.0001	*p*=0.1388
Small problem	2.64 (1.35–5.18)	0.95 (0.43–2.09)		
Some-big problem	17.12 (9.42–31.11)	12.5 (7.04–22.2)		
Cognition				
No problem (ref.)			*p*<0.0001	*p*=0.9875
Small problem	1.18 (0.63–2.22)	1.11 (0.56–2.2)		
Some problem	2.67 (1.45–4.9)	2.88 (1.62–5.15)		
Big problem	5.19 (1.77–15.18)	6.17 (2.15–17.67)		
Fatigue				
No problem (ref.)			*p*<0.0001	*p*=0.1775
Small problem	1.43 (0.68–3.02)	2.1 (0.92–4.77)		
Some problem	3.97 (1.99–7.9)	5 (2.32–10.77)		
Big problem	15.03 (5.17–43.73)	5.24 (1.8–15.27)		
Breathing				
No problem (ref.)			*p*=0.0011	*p*=0.6293
Small problem	1.64 (0.89–3.03)	1.53 (0.8–2.95)		
Some problem	2.81 (1.51–5.21)	2.48 (1.38–4.44)		
Big problem	1.41 (0.51–3.93)	3.24 (1.17–9.03)		
Hospitalisation	–	–	*p*=0.0021	*p*=0.0152

Analysed with a multivariate binary logistic regression for men and hospitalisation (yes/no) as both main effect and all pairwise interactions of all exploratory variables. Within ‘age groups’ and ‘grading of movement’, categories were collapsed due to insufficient quantity (<5). Odds ratio (OR) >1 indicates that the variable influences daily life activity. Sick leave >3 months, movement, cognition, fatigue, breathing and hospitalisation made a significant contribution to the model even after applying Bonferroni correction (10 tests, corrected significance level=0.005). Nagelkerke *R*^2^ 71%, correctly classified 94.6% of cases, 915 included, 63 missing.

CI: confidence interval, ref.: reference.

For women (Table IV), sick leave of more than three months, acute symptoms, movement and fatigue made a significant contribution to the model even after applying Bonferroni correction (8 tests, corrected significance level=0.00625). Hospitalisation (after adjusting for the other explanatory variables) did not show any statistically significant effect. Regressions presented without interaction, for men and woman respectively, are available in the supplemental material.

**Table IV.** Explanatory factors for problems with daily life activity among women.

	Non-hospitalised woman	Hospitalised woman	Association of the variable as such (in at least one of the subgroups)	Difference between groups
Age (years)				
18–30 (ref.)			*p*=0.1681	*p*=0.2421
31–40	1.6 (0.85–3)	1.16 (0.18–7.58)		
41–50	1.5 (0.84–2.69)	0.58 (0.11–3.02)		
51–60	1.53 (0.86–2.71)	1.31 (0.26–6.56)		
⩾60	1.07 (0.57–2.02)	0.96 (0.18–5.06)		
Education				
Primary school (ref.)			*p*=0.1720	*p*=0.0629
Secondary school	0.75 (0.39–1.41)	3.65 (1.14–11.69)		
Short university education	0.79 (0.39–1.58)	5.14 (1.43–18.43)		
Long university education	0.65 (0.34–1.24)	3.81 (1.16–12.54)		
Sick leave length >3 months	2.08 (1.34–3.23)	2.17 (1.15–4.1)	*p*=0.0002	*p*=0.9121
Acute symptoms				
No-moderate problems (ref.)			*p*<0.0001	*p*=0.0970
Quite severe problems	1.7 (1.3–2.24)	0.58 (0.22–1.54)		
Very severe problems	3.15 (2.07–4.78)	0.99 (0.37–2.65)		
Movement				
No problem (ref.)			*p*<0.0001	*p*=0.1314
Small problem	2.33 (1.62–3.33)	2.54 (1.21–5.33)		
Some-big problem	13 (9.62–17.57)	25.34 (13.15–48.82)		
Fatigue				
No problem (ref.)			*p*<0.0001	*p*=0.9316
Small problem	1.92 (1.22–3.03)	2.03 (0.86–4.79)		
Some problem	7.78 (5.3–11.4)	6.53 (3.2–13.32)		
Big problem	29.42 (18.23–47.48)	24.55 (9.3–64.79)		
Breathing				
No problem (ref.)			*p*=0.1312	*p*=0.8151
Small problem	0.97 (0.69–1.36)	0.78 (0.38–1.59)		
Some-big problem	1.41 (1.03–1.91)	1.15 (0.6–2.19)		
Hospitalisation	–	–	*p*=0.1962	*p*=0.2431

Analysed with a multivariate binary logistic regression for woman and hospitalisation (yes/no) as both main effect and all pairwise interactions with all exploratory variables. In the regression, sick leave prior to Covid-19 and cognition were excluded due to a correlation >0.7 with length of sick leave and grading of fatigue, respectively. Odds ratio (OR) >1 indicates that the variable influences daily life activity. Sick leave length >3 months, acute symptoms, movement and fatigue made a significant contribution to the model even after applying Bonferroni correction (8 tests, corrected significance level=0.00625). Hospitalisation (after adjusting for the other explanatory variables) did not show any statistically significant effect. Nagelkerke *R*^2^ 67.4%, correctly classified 93.3% of cases, 588 included, 28 missing.

CI: confidence interval; ref.: reference.

## Discussion

This nationwide study found that 85.6% of people who had been on sick leave due to Covid-19 during the first wave of the pandemic in Sweden experienced problems with body function 18 months after infection, and nearly half of them experienced problems with daily life activities. Fatigue was the most prevalent problem, followed by problems with cognition, sleep and movement. Furthermore, problems were generally more prevalent in women and among those who had initially been hospitalised.

In the current study, the reported prevalence of at least one problem with body function could be seen as remarkably high compared to studies with shorter follow-up [6–10,14]. That the prevalence of problems may increase over time could reflect individuals’ realisations of problems being more prolonged than anticipated [16]. Similarly, a Chinese study using face-to-face interviews, physical examinations and questionnaires in more than 1000 previously hospitalised (46% women) individuals reported an increase in symptoms between the one-year (49% reported at least one symptom) and two-year follow-up (55%) [15]. In the current study, the high prevalence of reported problems at 18 months may be partly explained by the larger proportion of women (66%) and the methodology, as people may be more inclined to answer a questionnaire if experiencing more problems. However, the prevalence reported in the current study is in line with a smaller Swedish study [21] with a two-year follow-up of previously hospitalised individuals exhibiting symptoms consistent with post-Covid condition at the four-month follow-up.

In the present study, different patterns of problems with body function were present, depending on initial Covid-19 severity and sex. Individuals who had initially been hospitalised generally reported a higher prevalence of problems. Coherently, previous studies have shown greater improvement for non-hospitalised individuals at the one-year follow-up [22] and a lack of recovery for hospitalised individuals 18 months after infection [13], as well as more severe disease being associated with the long-term consequences of Covid-19 [12,23]. Surprisingly, in regard to anosmia/ageusia, heart rhythm and temperature alterations, non-hospitalised individuals reported a higher prevalence of problems. However, these differences may be affected by the skewed population, with men accounting for 59% of hospitalised participants and women for 75.3% of non-hospitalised participants, as the largest sex differences were found in the same three body functions.

Women generally reported a higher prevalence of problems, which is in line with female sex previously being reported as a risk factor for long-term consequences of Covid [15,24] and being associated with lack of recovery up to 18 months after infection [13]. Surprisingly, there were no sex differences for problems with daily life activities. The mechanisms underlying these long-term consequences, as well as women’s susceptibility to them, remain unclear [25]. One small German study investigating the association between symptoms and immunological parameters at a 12-month follow-up found that symptoms were more prevalent in individuals with elevated antinuclear antibody (ANA) titres [26]. Furthermore, more women had elevated ANA titres, and when analysing sex separately, an association between elevated titres and symptoms was only found for women [26], suggesting that autoimmunity may partly explain why women tend to experience more long-term problems [26,27].

Sick leave of more than three months, movement and fatigue significantly contributed to whether individuals experienced problems with daily life activities in both woman and men (regression models). This indicates that individuals who experienced problems with fatigue and movement were significantly more likely to report problems with daily life activities than those who experienced no problems with fatigue and movement, regardless of sex and Covid-19 severity. The ORs for fatigue were higher for women than for men, which suggests that fatigue has a larger impact on daily life activities for women. The women within our cohort also had a higher reported prevalence of fatigue than the men, which confirms findings in the general population [28]. Sick leave of more than three months also plays an important role in managing daily activities for both men and woman. Even though the results are based on low numbers of hospitalised men and should therefore be interpreted with caution, this may indicate that the initial severity itself may be a significant contributor as to whether they experience problems with daily life activities, which is in line with hospitalisation being reported as the most prominent predictor of long-term consequences of Covid-19 [29]. However, this was not present for hospitalised women. Thus, the dynamics of the influencing factors are not yet clear.

Problems with breathing contributed to explaining whether men experienced problems with daily life activities. However, this was not a significant explanatory factor for women. Problems with breathing was also one of the few body functions for which sex differences were not prevalent, as reported elsewhere [26]. As men have been hospitalised more frequently due to Covid-19 [30], in line with the current cohort, with problems with breathing being an indication for hospital admission, this may partly explain why having problems with breathing seems to be a more relevant explanatory factor for men.

The models indicate some variations in explanatory factors 18 months post Covid-19 in men and women on daily life activities. The differences could be important when understanding the underlying factors that contribute to restrictions on an individual level, as well as in the treatment of different symptoms. This study incorporates a large study population: 11,955 individuals with 5464 responders from a nationwide study with a cohort compiled from the registries of a national institution. The results of this study are therefore robust and may create a picture of the long-term consequences people may face due to Covid-19 during the first wave. Although it was a large study population, only 45.7% responded, and responders significantly differed from non-responders in all investigated characteristics, including sex and Covid-19 severity. Responders were more likely to be women and hospitalised, both found to be associated with a higher prevalence of problems in the present study. Therefore, the results may be exposed to response bias, which, with the possibility of recall bias and the lack of a control group, could result in an overestimation of the prevalence of long-term consequences of Covid-19. Covering the early phase of the pandemic may also involve some uncertainty in the sick-leave data, as some individuals may have been misclassified as having Covid-19 when in fact they did not, and if so, this is probably overrepresented in the group of non-responders at 18 months. The results should be interpreted with this in mind. Further research should aim to explore the long-term consequences in people on sick leave during later phases of the pandemic, with a focus on the variety in symptoms and differences between groups. The duration of problems may also be of continued interest, considering different treatment options as well as rehabilitation to consequences.

## Conclusions

In this national cohort of individuals who were on sick leave due to Covid-19 during the first wave of the pandemic in Sweden, more than 8 out of 10 experienced problems 18 months later, with women and initially hospitalised individuals generally reporting more problems. The results indicate that problems with body function can partly explain problems with daily life activities. However, non-individual factors may also have an influence, and differences depending on sex and initial Covid-19 severity should be considered. This study contributes to the current knowledge of the persistent long-term consequences of Covid-19. However, the mechanisms behind the consequences are not yet clear and need to be further investigated.

## Supplemental Material

sj-docx-1-sjp-10.1177_14034948241272949 – Supplemental material for Self-reported body function and daily life activities 18 months after Covid-19: A nationwide cohort study Supplemental material, sj-docx-1-sjp-10.1177_14034948241272949 for Self-reported body function and daily life activities 18 months after Covid-19: A nationwide cohort study by Johanna Seljelid, Annie Palstam, Katharina S. Sunnerhagen and Hanna C. Persson in Scandinavian Journal of Public Health
