# Supplementary material for: Self-reported body function and daily life activities 18 months after Covid-19: A nationwide cohort study
Source: Scand J Public Health. 2024 Sep 18;53(5):465–73. doi: 10.1177/14034948241272949 (PMC12159346; doi:10.1177/14034948241272949)
Supplement: sj-docx-1-sjp-10.1177_14034948241272949 – Supplemental material for Self-reported body function and daily life activities 18 months after Covid-19: A nationwide cohort study [file sj-docx-1-sjp-10.1177_14034948241272949.docx]

**Appendix Table 1A: Regression model for men, without interaction.**

|  | OR (95% CI) | Association of the variable with the outcome | Holm-Bonferroni corrected p-values |
| --- | --- | --- | --- |
| **Age**, 18-40 (ref) |  | p = 0.3045 | Non-significant |
| 41-50 | 1.16 (0.65-2.08) |  |  |
| 51-60 | 1.53 (0.9-2.62) |  |  |
| >=60 | 1.2 (0.67-2.17) |  |  |
| **Education**, primary school(ref) |  | p = 0.3700 | Non-significant |
| Secondary school | 0.62 (0.36-1.06) |  |  |
| Short university education | 0.68 (0.37-1.27) |  |  |
| Long university education | 0.66 (0.36-1.21) |  |  |
| **Sick-leave prior to Covid-19** | 0.94 (0.57-1.54) | p =0.7993 | Non-significant |
| **Sick-leave length>3months** | 1.78 (1.17-2.73) | p=0.0075 | 0.0376 |
| **Acute symptoms**, no-moderate (ref) |  | p = 0.5164 | Non-significant |
| Quite severe problems | 1.11 (0.71-1.75) |  |  |
| Very severe problems | 1.33 (0.8-2.21) |  |  |
| **Movement**, no problem (ref) |  | p <.0001 | p <.0001 |
| Small problems | 1.63 (1-2.66) |  |  |
| Some-big problems | 13.92 (9.32-20.81) |  |  |
| **Cognition**, no problem (ref) |  | p <.0001 | p <.0001 |
| Small problem | 1.2 (0.77-1.88) |  |  |
| Some problem | 2.85 (1.9-4.28) |  |  |
| Big problem | 6.26 (3.02-12.99) |  |  |
| **Fatigue**, no problem (Ref) |  | p <.0001 | p <.0001 |
| Small problem | 1.65 (0.96-2.82) |  |  |
| Some problem | 4.14 (2.52-6.78) |  |  |
| Big problem | 8.07 (3.89-16.77) |  |  |
| **Breathing**, no problem (ref) |  | p =0.0002 | 0.0015 |
| Small problem | 1.53 (0.99-2.37) |  |  |
| Some problem | 2.49 (1.65-3.76) |  |  |
| Big problem | 2.13 (1.05-4.29) |  |  |
| **Hospitalisation** | 0.57 (0.39 - 0.84) | p=0.0039 | 0.0233 |

Comment: Analysed with a multivariate binary logistic regression for men. In the regression ‘sick-leave prior to Covid-19’ was excluded due to a correlation >0.7 with ‘length of sick leave’. Within ‘age groups’ and ‘grading of movement’ categories were collapsed due to insufficient quantity (<5). Odds ratio (OR) > 1 indicates that the variable influences daily life activity. After adjusting for all the other variables, hospitalization is protective and significant for men. After Holm-Bonferroni adjustment (10 tests, nominal level 0.05), all variables that were significant in the uncorrected model, remains significant.

**Appendix Table 1B: Regression model for woman, without interaction.**

|  | OR (95% CI) | Association of the variable with the outcome | Corrected p-values (Holm-Bonferroni) |
| --- | --- | --- | --- |
| **Age**, 18-30 (ref) |  | p = 0.2343 | Non-significant |
| 31-40 | 1.56 (0.86-2.83) |  |  |
| 41-50 | 1.35 (0.78-2.34) |  |  |
| 51-60 | 1.54 (0.9-2.63) |  |  |
| >=60 | 1.14 (0.64-2.05) |  |  |
| **Education**, primary school (ref) |  | p = 0.5968 | Non-significant |
| Secondary school | 1.04 (0.6-1.82) |  |  |
| Short university education | 1.18 (0.64-2.16) |  |  |
| Long university education | 0.93 (0.53-1.65) |  |  |
| **Sick-leave prior to Covid-19** | 1.09 (0.82-1.44) | p=0.5580 | Non-significant |
| **Sick-leave length >3months** | 2.03 (1.42-2.89) | p = 0.0001 | 0.0005 |
| **Acute symptoms**, no-moderate (ref) |  | p <0.0001 | p <.0001 |
| Quite severe problems | 1.61 (1.24-2.09) |  |  |
| Very severe problems | 2.76 (1.93-3.94) |  |  |
| **Movement**, no problem (ref) |  | p <.0001 | p <.0001 |
| small | 2.4 (1.74-3.31) |  |  |
| Some-big | 14.54 (11.12-19.03) |  |  |
| **Fatigue**, no problem (Ref) |  | p <.0001 | p <.0001 |
| Small problem | 1.91 (1.29-2.84) |  |  |
| Some problem | 7.22 (5.18-10.08) |  |  |
| Big problem | 26.69 (17.51-40.68) |  |  |
| **Breathing**, no problem (ref) |  | p = 0.0315 | 0.1574 |
| Small problem | 0.94 (0.7-1.28) |  |  |
| Some-big problem | 1.37 (1.04-1.8) |  |  |
| **Hospitalisation** | 0.80 (0.59 - 1.08) | p= 0.1423 | Non-significant |

Comment: Analysed with a multivariate binary logistic regression for woman. In the regression ‘sick-leave prior to Covid-19’ and cognition was excluded due to a correlation >0.7 with ‘length of sick leave’ and ‘grading of fatigue’, respectively. Odds ratio (OR) > 1 indicates that the variable influences daily life activity. Sick leave length > 3 months, acute symptoms, movement, and fatigue made a significant contribution to the model even after applying Holm-Bonferroni adjustment (8 tests, nominal level 0.05). Hospitalisation (after adjusting for the other explanatory variables) did not show any statistically significant effect.
